# Unusual Cause of Acute Scrotal Pain-Inflammatory Noncommunicating Hydrocele: A Pediatric Case Report

**DOI:** 10.1155/2018/2862514

**Published:** 2018-02-08

**Authors:** Yoshinobu Moritoki, Kentaro Mizuno, Taiki Kato, Takahiro Yasui, Yutaro Hayashi

**Affiliations:** ^1^Department of Nephro-Urology, Nagoya City University Graduate School of Medical Sciences, Nagoya, Japan; ^2^Department of Pediatric Urology, Nagoya City University Graduate School of Medical Sciences, Nagoya, Japan

## Abstract

The etiology of scrotal pain is clinically classified in terms of the necessity for emergency surgery. Lately, color Doppler ultrasonography has reduced unnecessary surgeries, but there are still some cases that require immediate exploration because of an uncertain diagnosis. Here, we describe the case of a 14-month-old boy, who could not deliver his complaint accurately, presenting with a grumpy mood and a red swollen scrotum. Emergency surgery revealed that the cause was intense inflammation of the hydrocele wall, which typically does not cause acute scrotum. We also reviewed rare etiologies of scrotal pain for general physicians to develop the differential diagnosis.

## 1. Introduction

Acute scrotal pain includes some urgent diagnoses, such as testicular torsion and incarcerated hernia or omentum. Traditionally, an in-depth interview and color Doppler ultrasonography (US) can lead to an acute diagnosis, although some cases exist where clinicians cannot eliminate emergency etiologies because the case history and physical examination are not performed accurately or the US revealed equivocal imaging. Here, we present a case where a presurgical diagnosis compromised incarcerated omentum, and the operation revealed that intense hydrocele inflammation was the cause of the symptoms.

## 2. Case Presentation

**Figure 1:**
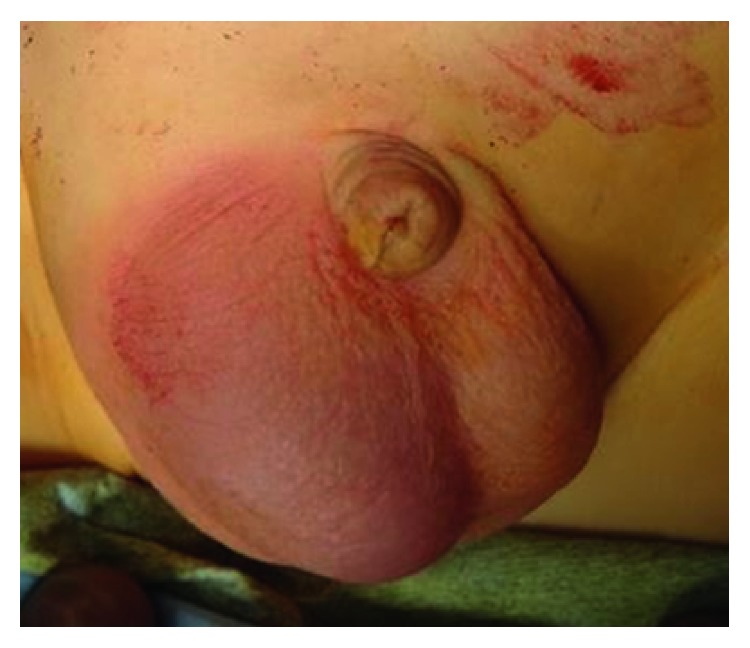
The scrotum was red and swollen.

**Figure 2:**
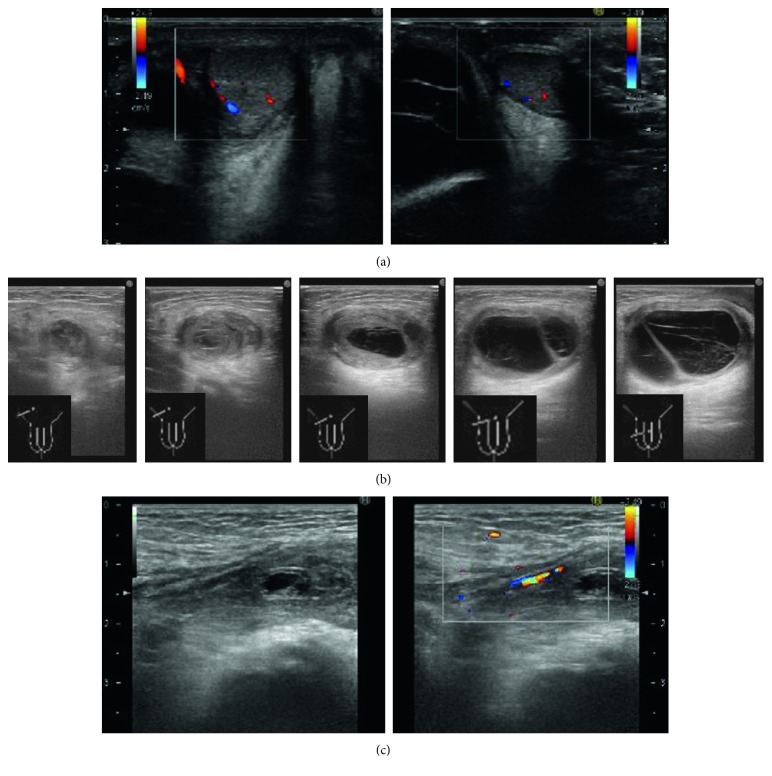
(a) Both testes were normal sized with normal blood flow. (b) The hydrocele wall was as thick as 3–5 mm (scale bar = 1 cm). (c) The omentum is inserted into the right inguinal canal. The blood flow was detected.

**Figure 3:**
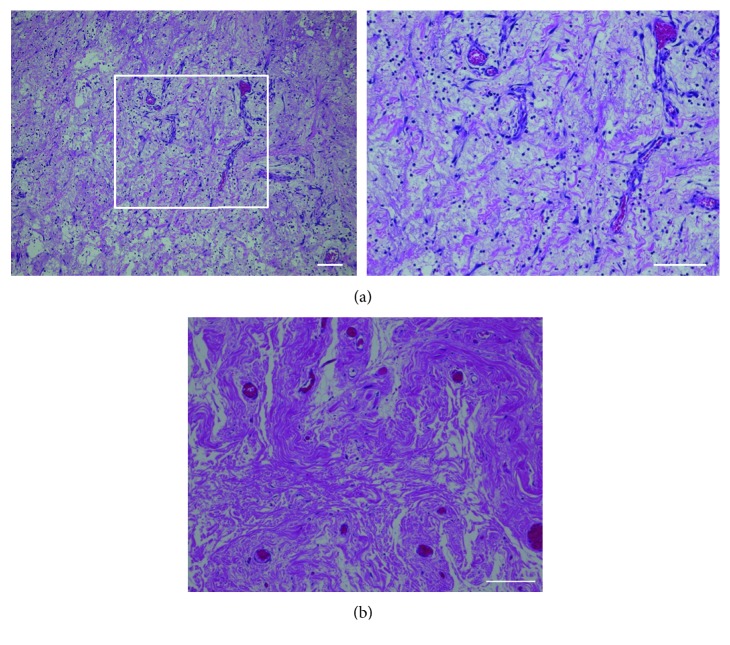
(a) The stroma was thick and edematous with fibrin precipitation and lymphatic cell infiltration. (b) Pediatric noncommunicating hydrocele aged at 1 year as a control. The tissue was not as edematous as in our case, and less lymphatic cells were infiltrated (scale bar = 100 *µ*m).

A 14-month-old boy who had been diagnosed with a hydrocele in the right spermatic cord was referred to our department from the outpatient clinic with a right swollen scrotum. He presented to our hospital for the management of intermittent right scrotum pain for 4 days. He was in grumpy mood, his right scrotum was red and swollen, and the cremasteric reflux was absent (Figure 1). The urine test showed no white blood cells, and blood tests showed a slight increase in CRP (1.27 ng/mL). The US showed normal testes and epididymis with normal blood flow by the color Doppler US (Figure 2(a)). On the cranial of the right testis, low-echoic hydrocele with multiseptum was present, and its capsulizing wall was as thick as 3–5 mm (Figure 2(b)). In the right inguinal canal, omentum was present in the patient's processus vaginalis with blood flow by the color Doppler US (Figure 2(c)). However, we could not completely rule out the partial omental incarceration considering the sensitivity of US, and thus, emergent operative intervention was performed. Under inguinal exploration, processus vaginalis (PV) was present with the omentum inserted, but there was no evidence of necrotic or adherent tissue. PV was not communicating with hydrocele. Surgical findings did not indicate that the omentum itself was the cause of scrotal pain, and we next went on to the testis exploration. Upon opening the right tunica albuginea, the testis was normal colored, and the spermatic cord did not experience torsion. However, the hydrocele wall was ubiquitously adherent with the surrounding tissue and was much thicker and more solid than typical pediatric testicular hydrocele. The enlarged hydrocele wall was removed, and its edges were oversewn in the surgery. Pathology of the hydrocele wall showed edema in the wall, fibrin precipitation, and lymphocyte infiltration (Figure 3(a)), indicating that severe inflammation caused scrotal pain and redness.

## 3. Discussion

Acute scrotum is characterized by scrotal pain that has rapid or acute onset. The most serious condition in pediatrics is testicular torsion that requires emergent surgery to avoid testicular necrosis. As the sensitivity of the US against testicular torsion is as high as 88.9–100% [1, 2] and the US in this case showed clear blood flow into both testes, we had to rule out another disease that required emergent surgery—incarcerated intestine or omentum into the inguinal canal. Even the highest sensitivity of the US for incarcerated inguinal hernia is 91% [3], and thus, the possibility of an incarcerated hernia could not be eliminated. Therefore, we proceeded to emergent surgical intervention.

Although the accurate etiology is uncertain, there are three reasons that suggest the painful red scrotum was derived from inflammation of hydrocele wall. First, the pathology findings revealed a thick hydrocele wall, a high number of inflammatory cells, and firm fibrin precipitation, those of which are not usually seen in typical pediatric hydroceles (Figure 3(b)). Second, there were no other differential diagnoses that could explain the cause of the pain. The other possible etiology was compression of the testicular vessels by a massive hydrocele or edematous incarcerated inguinal hernia [4]. These conditions are rare, with only five cases being reported to date [4–8]. In each of those cases, the preoperative US clearly showed reduction in testicular blood flow. Based on these findings, the effect of compression could be ruled out in our case. The third observation is the type of onset. Acute scrotum pain due to ischemia is caused by vessel obstruction; therefore, the onset should be sharp and sudden. In the present case, the patient was referred to our department after 4 days of pain initiation. Retrospectively, the time course suggests that the cause is not likely to be ischemic, although we could not be certain that the testis or omentum was intact. All these observations suggest that the cause of the pain was inflammatory changes in the hydrocele.

The reason for hydrocele inflammation is uncertain. It is widely known that incarcerated hernias sometimes cause hydroceles. In our case, however, there was no ischemia of the omentum; nor was the hydrocele communicating with abdominal cavity, suggesting other reasons exist for hydrocele inflammation. Clinically, it was reported that omentum often form adhesions to inflamed regions such as the patient's PV [3]. In our case, possibly, omentum caused inflammatory changes in the PV and the inflammation spread to the hydrocele wall, although this is just a speculation. Pediatric hydroceles are common and often asymptomatic. To our knowledge, this is the first case of a pediatric hydrocele that caused acute scrotum pain. In retrospect, surgery was not necessary in this case; the intention was to ensure that we were ruling out tissue ischemia, a critical condition. Physicians should not hesitate to perform emergency surgery if the cause of scrotal pain cannot be determined with confidence.
